# Surgical simulation training should become a mandatory part of orthopaedic education

**DOI:** 10.1186/s40634-022-00455-1

**Published:** 2022-02-28

**Authors:** Romain Seil, Claude Hoeltgen, Hervé Thomazeau, Hermann Anetzberger, Roland Becker

**Affiliations:** 1 Sports Clinic, Centre Hospitalier de Luxembourg – Clinique d’Eich, 8 rue d’Eich, 1460 Luxembourg, Luxembourg; 2 Luxembourg Institute of Research in Orthopaedics, Sports Medicine and Science, Luxembourg, Luxembourg; 3 Human Motion, Orthopaedics, Sports Medicine and Digital Methods, Luxembourg Institute of Health, Strassen, Luxembourg; 4 VirtaMed AG, Rütistrasse 12, CH-8952 Schlieren, Switzerland; 5 Univ Rennes, INSERM, LTSI - UMR 1099, 35000 Rennes, France; 6 Orthopädische Gemeinschaftspraxis am OEZ, Hanauer Str. 65, 80993 Munich, Germany; 7 Department of Orthopaedics and Traumatology, University of Brandenburg an der Havel, Hochstrasse 29, 14770 Brandenburg/Havel, Germany

**Keywords:** Simulation, Training, Education, Arthroscopy, Surgery, Curriculum, Competencies

## Abstract

**Purpose:**

Ethical concerns and increasing economic constraints of hospitals have caused a reduction in proper training and education. It has been hypothesized that due to the lack of a one-to-one apprenticeship throughout the residency, surgical simulation training is essential.

**Methods:**

Between June 2020 and June 2021, residents from teaching hospitals in Switzerland, France, Germany, and Luxembourg were surveyed to learn about their experience with and thoughts on surgical simulation training. Survey responses were analysed using descriptive statistics.

**Results:**

Of the 596 residents surveyed, 557 residents (51% female, 49% male) from Switzerland (270), France (214), Germany (52) and Luxembourg (21) agreed to anonymous data analysis. Among those giving consent, 100% considered that simulation training was important for their practical education and 84% thought that simulation training should become a mandatory part of their curriculum, with an average estimated training time of 42 ± 51 h per year, based on the survey.

**Conclusions:**

This study suggests that surgical simulation training is well accepted and even demanded among surgical residents as an alternative training solution able to address some of the limitations and challenges of the current one-to-one apprenticeship model. There is a wide variation among the residents regarding the number of training hours required, underscoring the need for structured performance-based simulator training.

## Introduction

Contemporary training of surgical competencies is still relying on principles of the nineteenth Century [8]. Yet, practical training opportunities corresponding to the present high levels of specialization in orthopaedic surgery are limited and expensive [7]. As the field of arthroscopy advances, it leaves little doubt that the one-to-one apprenticeship model throughout the residency training is no longer sustainable and that the fundamental structure of surgical education is at a turning point [6]. Due to the complex requirements, learning arthroscopic skills is inevitably associated with a comparatively longer operation time and a higher rate of intraoperative complications [15]. Reduced working-time directives, ethical concerns and the present challenges linked to the COVID-19 global pandemic have only reinforced the need for access to alternative surgical training solutions [9, 17].

According to Fitts and Posner’s well accepted three-stage theory of motor skills acquisition [20], the earlier stages of teaching technical skills should take place outside the operating room until automaticity in basic skills is achieved, and trainees can focus on more complex issues in the operating room. Already in 2008, researchers in Oxford, England demonstrated that motor skills acquired on an arthroscopic knee simulator could be effectively transferred into the operating room [10]. Hence, simulation-based training is an important steppingstone in surgical education as it allows residents to reach sufficient levels of motor skills in a risk-free environment to minimize clinical morbidity, optimize surgical resources, and maximize their training experience [21]. Indeed, a recent study concluded that knee arthroscopy simulation training with self-learning modules can improve patient safety overall, as residents become more skilled earlier in their training, leaving more time for their mentor to teach advanced skills [5].

Although simulation training has been part of medical education in different forms for hundreds of years, arthroscopic surgery has a far higher degree of complexity, thus requiring more training hours before achieving proficiency [14]. An important difficult step in arthroscopy training is to achieve the skills of triangulation, which means bringing together the angled optics and the instruments within a joint [19]. Excellent hand-eye coordination as well as spatial imagination is required, as the three-dimensional space of the joint is represented as a 2D image on the screen. This particularity of arthroscopic surgery makes virtual reality simulation training through a 2D monitor especially well-suited to teaching arthroscopy skills, compared to 3D immersive virtual reality technologies and other types of simulation training. In general, simulation training allows disassembling the complex requirements of arthroscopic surgeries into simple elementary steps which can be trained without harming the patients.

It has been hypothesized that, due to the challenges and limitations of the current surgical training and education models and the benefits associated with simulation training, residents request surgical simulation training to be included in their residency training curriculum.

## Methods

### Survey development

The survey was designed from a resident’s perspective as a tool to study whether surgical simulation training is requested and should be included as an integral part of the residency program in Orthopaedics and Traumatology. The survey questions were developed based on previous experience regarding residency training and education [11, 24]. Participants were residents from teaching hospitals in Switzerland, France, Germany, and Luxembourg. An electronic survey was answered via an online survey tool (Google Forms) after an individual practice session of 1 h with a high-fidelity surgical simulator (VirtaMed) between June 2020 and June 2021 (Table 1). Consent by the participants was required for their anonymized data to be included in the analysis.

**Table 1:** Questions that residents were asked via an online survey tool in the local language or in English respectively. The first two questions were mandatory while the last question was optional

French questionnaire	German questionnaire	English questionnaire
Trouvez-vous la formation par simulation utile?	Erachten Sie Simulationstraining als sinnvoll?	Do you find simulation training useful?
Souhaiteriez-vous que la formation par simulation soit obligatoire?	Wünschen Sie, dass Simulationstraining Pflicht wird?	Do you wish simulation training to be mandatory?
Combien d’heures par année souhaiteriez-vous obligatoires?	Wie viele Stunden Simulationstraining pro Jahr würden Sie als verpflichtend wünschen?	How many hours per year should be mandatory?

### Data analysis

Data was retrieved from the online survey database and summarized. Standard descriptive analytics were performed to analyse extracted data. Since not all values were normally distributed, non-parametric tests were used for the statistical analysis. Kruskal-Wallis test with post hoc Mann-Whitney test was used to determine significant differences between the countries. Probability values less than 0.05 were considered significant. Statistical analysis was performed using SPSS, Version 26.0 (IBM, New York, USA).

## Results

Of the 596 residents surveyed, 557 residents (51% female, 49% male) agreed that their data was being used for anonymous data analysis. Of the 557 residents, 270 were doing their residency training in Switzerland (49%), 214 in France (38%), 52 in Germany (9%) and 21 in Luxembourg (4%). In summary, 100% of residents considered that simulation training is important for their practical education and 84% thought that simulation training should become a mandatory part of their curriculum (Table 2). On average, residents indicated a surgical simulation training time of 42 ± 51 h per year as a relevant compulsory allotted training time during residency.

**Table 2:** National trends in residents’ surgical simulation perception

Country of residency	Number of residents (n) surveyed	Residents (%) finding simulation training useful	Residents (%) in favour of mandatory simulation training	Number of residents (n) specifying mandatory training hours	Declared training time in hrs / y mean ± SD (median, min-max)
Switzerland	270	100%	88%	232	36 ***±*** 52 (20, 1–450)
France	214	99%	79%	60	63 ***±*** 48 (48, 1–192)
Germany	52	100%	87%	43	47 ***±*** 46 (36, 2–180)
Luxembourg	21	100%	86%	18	40 ***±*** 40 (30, 1–150)
total	557	100%	84%	353	42 ***±*** 51 (24, 1–450)

**Fig. 1:**
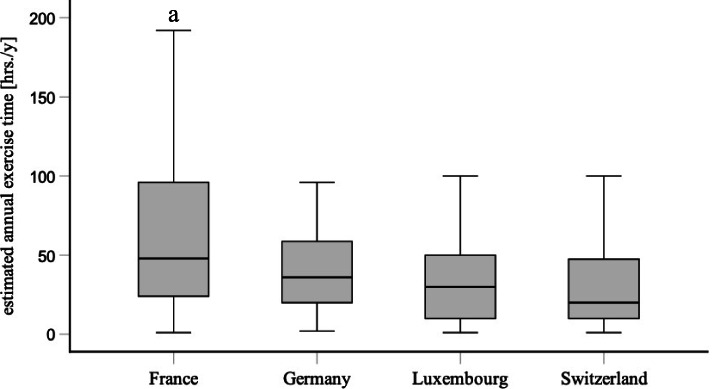
Mandatory surgical simulation training times. Boxplot showing annual surgical simulation training hours estimated by residents in Switzerland, France, Germany, and Luxembourg as an appropriate mandatory workload within their residency curriculum. The interquartile range (IQR) is represented by the box, the median is represented by the line in the box, IQR * 1.5 is represented by the whiskers. ^a^ *p* < 0.05 France vs Germany, Luxembourg, and Switzerland

Residents in France were slightly less convinced that surgical simulation training should become mandatory (79%) compared to those in Switzerland (88%), Germany (87%) and Luxembourg (86%). In contrast, the residents in France suggested on average more mandatory training hours (62 ± 48 h.) annually in comparison to their peers in Switzerland (36 ± 52 h.), Germany (47 ± 46 h.) and Luxembourg (40 ± 40 h.). A wide range in suggested mandatory training times was observed (Fig. 1). 23 residents even demanded more than 120 h of simulation training annually.

## Discussion

The most important finding of this survey was the need of surgical simulation training requested by residents in Switzerland, Germany, France, and Luxembourg. Almost all participants found the virtual reality training beneficial for the development of their skills, and the vast majority even demanded it to become an integral part of the residency training curriculum, with an approximate average mandatory training time of 42 h per year. The findings on surgical simulation training in this study are based on an experience with virtual reality simulators. In accordance with the outcomes of our study, 80% of orthopaedic surgery program directors in the USA also believed that surgical skills simulation should be a mandated component of surgical training [11].

Surprisingly, compared to doctors in Switzerland, Germany and Luxembourg, residents in France were slightly less decided of having surgical simulation training as a mandatory part of their residency training. Yet, since November 2017, every surgical resident in France has to train some time on a simulator prior to operating on patients [26]. It was the French College of Orthopaedic Surgery and Traumatology (CFCOT) in collaboration with the Francophone Society of Arthroscopy (SFA) and a medical simulation company that collaborated on a two-part nationwide study in arthroscopic training methods to determine how best to meet this mandate for orthopaedics [25].

While surgical simulation training is not mandated in Switzerland, however an arthroscopy simulator is used in the annual orthopaedic certification exam since 2013 to evaluate how the candidates handle tools, how they navigate in the joint, and how they visualize the relevant anatomic landmarks. The AGA-Society for Arthroscopy and Joint Surgery regularly organizes the intensive 2-days STArt (**S**imulator **T**raining **Art**hroscopy) courses, where young orthopaedic trainees are taught by experienced arthroscopists through a set of theoretical and practical sessions on how to perform arthroscopies [3]. For the practical part of the course, an arthroscopy simulator is used. Based on these courses, the AGA instructors developed the Diagnostic Arthroscopic Skill Score (DASS) to reliably evaluate a trainee’s competency for performing diagnostic knee arthroscopies using a simulator [2]. The scoring system can support the implementation of proficiency-based training on the simulator. In another study, the same authors concluded that trainees can effectively learn arthroscopic skills on a simulator but that, based on the DASS, 10 hours of simulation training are insufficient to reach the target level set by experienced arthroscopists [4]. Furthermore, a one-off training session does not seem to be as successful as a training with repetition after some intervals [25].

Interestingly, the French residents requested a significantly higher number of practice hours compared to the other countries. The reason for this could be that simulator training for students and residents has already been introduced nationwide in France. Therefore, based on their experience, trainees are better able to estimate that many practice hours are required to perform adequately.

The aim of developing and implementing any curriculum including simulation training is to produce well trained and confident surgeons while safeguarding and improving patient safety [12]. It has been suggested that approximately 170 arthroscopic procedures are necessary to internalize the skills required to perform arthroscopic surgery [18]. This supports the concept of a structured inclusion of simulation training in existing curricula, given that the number of arthroscopies recommended to achieve basic competence regularly exceeds the minimum number required for residency training in some countries [13].

Alongside the push for additional simulation training, there has been a push toward new forms of curriculum, specifically proficiency-based progression [6] or competency-based progression [16]. Previously, training was based upon the amount of time in a rotation and not on the levels of skills acquired. This is inconsistent with the fact that innate arthroscopic skills and the amount of time to reach proficiency varies widely between trainees [1], which in return can explain part of the high range in the simulation training hours suggested by the surgical residents in this study. Hence, a growing turn towards educational efficacy and efficiency is pressuring training programs to demonstrate clinical proficiency using impartial assessment tools and encourage educational models which emphasize deliberate practice over the less-effective approach of repeated practice. If used appropriately and embedded into a structured training curriculum, simulation training is perfectly adapted to respond to these needs.

It falls within the responsibilities of local medical authorities as well as professional and scientific medical societies to set recommendations and expectations for practitioners and institutions [23]. Therefore, these societies are in the unique position to support the change management necessary to implement new training standards and spearhead the creation of a simulation-inclusive curriculum as part of modern surgical training. The European Society of Sports Traumatology, Knee surgery and Arthroscopy (ESSKA) has recently developed a competency-based core curriculum divided by anatomical region with the aim to describe a set of treatment core competencies [22]. Consequently, there is currently an opportunity for national professional orthopaedics societies to build on these recommendations by similarly defining standards for the use of simulation training in surgical education, especially in the field of arthroscopy. This should also include the development of curriculum guidelines that outline core content, objectives, and provide for adequate and impartial assessment of skills. More countries should be included in the assessment, which is currently a limitation of the study. Due to the introduction of the DASS, training can be performed in a highly standardised manner. It will allow also to underline potentially the results which are currently based solely on a few countries of Europe.

## Conclusions

This study suggests that surgical simulation training is well accepted and even demanded among surgical residents as an alternative training solution able to address some of the limitations and challenges of the current one-to-one apprenticeship model. In average 42 h per year of surgical simulation training is estimated appropriate by residents to become mandatory in the residency training curriculum.

## Data Availability

All data generated or analyzed in this study are included in this published article.
